# Spiritual Writings

**Published:** 1852-03

**Authors:** 


SPIRITUAL WRITINGS.

The northern humbug has taken another shape, and "spiritual rappings" are about to be superseded by "spiritual writings." The excitement growing out of this new phase of the superstition is leading, as might be foreseen, to the most deplorable consequences. Many of the victims of the delusion are said to have been driven mad. These "spiritual writers," who are very numerous and rapidly increasing in some of the northern States, profess to be able to communicate directly and familiarly with the spirits of the departed, and even with the Supreme Being himself; and, strange as it appears to us, there are multitudes who are duped by the monstrous imposition. The writers, in many cases, are evidently self-deluded. Under the influence of a species of hysteria, their hands execute movements which their wills seem hardly to dictate, and words which are in their minds are written apparently without a voluntary effort. The thing seems to them miraculous; they are confounded; reason and common sense are surrendered, and they give themselves over to the wildest extravagances.

We have many analogies for the phenomena in the history of medical superstitions, to which it may be worth while to refer on a future occasion.

